# Student Engagement in Mathematics Flipped Classrooms: Implications of Journal Publications From 2011 to 2020

**DOI:** 10.3389/fpsyg.2021.672610

**Published:** 2021-05-31

**Authors:** Chung Kwan Lo, Khe Foon Hew

**Affiliations:** ^1^Department of Mathematics and Information Technology, The Education University of Hong Kong, Hong Kong, China; ^2^Faculty of Education, The University of Hong Kong, Hong Kong, China

**Keywords:** flipped classroom, flipped learning, mathematics education, literature review, systematic review

## Abstract

Mathematics is one of the core STEM (science, technology, engineering, and mathematics) subject disciplines. Engaging students in learning mathematics helps retain students in STEM fields and thus contributes to the sustainable development of society. To increase student engagement, some mathematics instructors have redesigned their courses using the flipped classroom approach. In this review, we examined the results of comparative studies published between 2011 and 2020 to summarize the effects of this instructional approach (vs. traditional lecturing) on students’ behavioral, emotional, and cognitive engagement with mathematics courses. Thirty-three articles in K–12 and higher education contexts were included for analysis. The results suggest that the use of the flipped classroom approach may increase some aspects of behavioral engagement (e.g., interaction and attention/participation), emotional engagement (e.g., course satisfaction), and cognitive engagement (e.g., understanding of mathematics). However, we discovered that several aspects (e.g., students’ attendance, mathematics anxiety, and self-regulation) of student engagement have not been thoroughly explored and are worthy of further study. The results of this review have important implications for future flipped classroom practice (e.g., engaging students in solving real-world problems), and for research on student engagement (e.g., using more objective measures, such as classroom observation) in mathematics education.

## Introduction

In recent years, careers in STEM (science, technology, engineering, and mathematics) fields have been growing rapidly. It is thus important to the sustainable development of society to improve students’ knowledge of STEM and to prepare human resources for STEM careers. Among the four subject disciplines, however, mathematics can be particularly frustrating (Moliner and Alegre, 2020) and can prevent students from pursuing their STEM major (Adams and Dove, 2016). For example, Gundlach et al. (2015) observed that students may have significant anxiety related to courses in statistics. Dove and Dove (2015) cautioned that negative learning experiences can lead to students avoiding mathematics and can even result in mathematics anxiety. As Van Sickle (2016) concluded, engaging students in mathematics courses can not only provide a solid foundation for their future studies but also help retain students in STEM fields.

Student engagement is important for learning, as high levels of engagement are associated with various desirable outcomes, such as higher levels of academic achievement and lower dropout rates (Fredricks et al., 2004; Terrenghi et al., 2019; Abín et al., 2020). To increase student engagement, some mathematics instructors (e.g., Wilson, 2013; Cronhjort et al., 2018; Lo and Hew, 2021) have redesigned their traditional lecture-based courses using the flipped (or inverted) classroom approach. Under this instructional approach, “events that have traditionally taken place inside the classroom now take place outside the classroom and vice versa” (Lage et al., 2000, p. 32). In some mathematics flipped classrooms, instructors present basic materials before class using instructional videos (Lo et al., 2017; Yang et al., 2019). Class time can thus be freed up for more instructor–student and student–student interactions (Lo et al., 2017; Bond, 2020; Erbil, 2020).

Although the results of recent meta-analytic reviews (e.g., Lo et al., 2017; Cheng et al., 2019) generally suggest that the use of the flipped classroom approach should increase students’ mathematics achievement compared to traditional lecturing, we currently know little about its effect on student engagement. To address this knowledge gap, Bond (2020) conducted a systematic review of studies of flipped classrooms in K–12 research. She found that this instructional approach overwhelmingly supported student engagement across studies. It is worth noting, however, that many of her included studies did not employ a comparative between-subjects research design (i.e., they were not quasi-experiments). Is the flipped classroom approach indeed superior to traditional lecturing in terms of student engagement? This question remains unanswered.

This review aims to summarize the effect of the flipped classroom approach on student engagement compared to traditional lecturing. We focus on mathematics, as it is one of the core STEM subject disciplines. More importantly, engaging students in learning mathematics helps retain students in STEM fields and thus contributes to the sustainable development of society. Therefore, the overarching goal of this review is to make suggestions for future practice of the flipped classroom approach and for research on student engagement in mathematics education. Using a three-dimensional (i.e., behavioral, emotional, and cognitive) model of student engagement, as defined by Fredricks et al. (2004), the following research questions (RQ1 to RQ3) are used to guide this systematic review.

RQ1: How does the flipped classroom approach influence students’ behavioral engagement compared to traditional lecturing?
RQ2: How does the flipped classroom approach influence students’ emotional engagement compared to traditional lecturing?
RQ3: How does the flipped classroom approach influence students’ cognitive engagement compared to traditional lecturing?

## Developing the Conceptual Framework for Research Synthesis

The conceptual framework for the research synthesis of this review is developed in two stages. First, we define the flipped classroom approach and traditional lecturing in mathematics education. The definitions help to establish the context for this review and enhance the consistency of the selection of studies. Second, we use the work of Fredricks et al. (2004) to define the framework for student engagement. This framework can serve as a lens to analyze the results of student engagement across studies.

### Traditional and Flipped Classrooms in Mathematics Education

To establish the context for this review, we first clarify the meaning of traditional and flipped classrooms. Despite the absence of an explicit definition of a traditional classroom, we can identify some common practices in mathematics education. For example, Dove and Dove (2015) described the traditional classroom as: (1) “Class primarily consists of teacher-directed lecture,” (2) “Most student practice occurs outside of class and individually,” and (3) “Most group work, if any, occurs outside the classroom” (p. 169). Consistent with other studies, mathematics instructors would first introduce students to course materials inside the classroom, where active learning techniques such as pair work and group discussion (e.g., Loux et al., 2016; Lo and Hew, 2020; Wang et al., 2020) are used occasionally. Students are then provided with a few in-class learning tasks, followed by homework to be done after class (e.g., DeSantis et al., 2015; Guerrero et al., 2015; Wasserman et al., 2017).

In contrast, students in mathematics flipped classrooms would first be introduced to course materials before class and then complete individual and/or group learning activities inside the classroom (Lo et al., 2017; Yang et al., 2019). It is important to note that some scholars have suggested that video lectures must always be used as the pre-class instructional medium in the flipped classroom approach (Bishop and Verleger, 2013). However, other scholars disagree. For example, He et al. (2016) commented that “qualifying instructional medium is unnecessary and unjustified” (p. 61). They supported their stand using the quasi-experimental study by Moravec et al. (2010), in which the researchers showed that the use of either pre-class videos or pre-class readings with worksheets is equally effective.

In this review, we believe that mathematics instructors would choose the best instructional medium, which may not necessarily be video, to deliver their course materials. Indeed, the Flipped Learning Network (2014) does not add such a constraint (i.e., the instructor must use video lectures before class) when defining the flipped classroom approach. Without qualifying the use of instructional medium, their definition emphasizes the instructional sequence of using the individual learning space for direct instruction (pre-class) and the resulting group learning space for interactive activities involving the application of knowledge (in-class). The definition has been used to define the flipped classroom approach in mathematics education (e.g., Heuett, 2017; Hodgson et al., 2017). Therefore, this review uses the conceptual definition offered by the Flipped Learning Network (2014) and does not impose additional constraints on instructional media or activities with respect to either pre- or in-class learning components.

### Student Engagement

According to Fredricks et al. (2004), student engagement consists of three dimensions, namely behavioral, emotional, and cognitive engagement. These three dimensions cover more or less everything in educational settings, as this three-dimensional model was established through a large-scale research synthesis (see Fredricks et al., 2004 for a review). Recently, the model has been adopted in the development of the flipped classroom approach (e.g., Bond, 2020; Lo and Hew, 2021). Therefore, the work of Fredricks et al. (2004) can offer a solid foundation for the research synthesis in this review.

First, behavioral engagement is concerned with students’ participation, effort, and conduct (Fredricks et al., 2004). Students with high behavioral engagement will be involved in school-related activities and make an effort to complete learning tasks (e.g., quizzes and homework). Moreover, they will follow school rules and classroom norms (i.e., positive conduct) and engage in minimal disruptive behavior. According to Bond (2020), the use of the flipped classroom approach increased students’ interaction and participation because the instructors were better able to utilize their class time to create an interactive learning environment. However, some undesirable behaviors were observed in flipped classrooms, such as skipping classes and unpreparedness for pre-class learning tasks (Heuett, 2017; Lo et al., 2017; Bond, 2020). In other words, the use of the flipped classroom approach can have either a positive or a negative impact on students’ behavioral engagement.

Second, emotional engagement is related to students’ affective reactions (e.g., interest, satisfaction, feelings, and anxiety) and attitudes toward or value placed on learning (Fredricks et al., 2004). Ideally, instructors should create a learning environment that can induce positive feelings in students and reduce their anxiety about learning (Hernández-Barco et al., 2021). Meanwhile, the learning tasks should promote student interest in course materials. Bond (2020) found that enjoyment was a frequently mentioned finding in research on flipped classrooms in the K–12 context. Students enjoyed learning using instructional videos (e.g., Khan Academy and screencasts by their teachers). Moreover, video lecturing may reduce students’ anxiety because they are able to re-watch the videos to better understand course materials before class (Bond, 2020). However, the findings from a review examining 22 studies indicated no significant difference in terms of student satisfaction in traditional and flipped classrooms (van Alten et al., 2019). Besides that, student emotional engagement in other aspects (e.g., interest) has not yet been examined.

Third, cognitive engagement is concerned with students’ level of investment in learning and self-regulation (Fredricks et al., 2004). Students’ investment in learning goes beyond the behavioral level and can be reflected in their preference for challenges (Connell and Wellborn, 1991). Newmann et al. (1992) further emphasized the inner psychological quality of cognitive engagement and students’ psychological investment in understanding and mastering course materials instead of simply completing their learning tasks. Therefore, how students regulate (e.g., plan, monitor, and evaluate) their learning is also related to cognitive engagement (Pintrich and de Groot, 1990; Zimmerman, 1990). Bond (2020) found that some flipped classroom interventions may support students’ self-efficacy and self-regulation, indicating increased cognitive engagement. However, as far as we know, no research synthesis has yet been published examining how the flipped classroom approach influences students’ cognitive engagement compared to traditional lecturing.

## Method

### Search Strategies

The process for selecting relevant studies followed the preferred reporting of items for systematic reviews and meta-analyses (PRISMA) statement (Moher et al., 2009). Five electronic databases were searched: (1) Academic Search Ultimate, (2) British Education Index, (3) Education Research Complete, (4) ERIC, and (5) Web of Science. The search string with relevant keywords and Boolean operators was as follows: (flip^∗^ OR invert^∗^) AND (class^∗^ OR learn^∗^ OR course^∗^) AND (math^∗^ OR algebra OR trigonometry OR geometry OR calculus OR statistics). The asterisk was used as a wildcard to include most of the common expressions of the flipped classroom approach (e.g., flipped classroom, flipped learning, inverted classroom, and inverted learning) in mathematics education (i.e., general mathematics and the three major content area – algebra, calculus, and statistics), such as a flipped learning algebra course (Murphy et al., 2016) and flipping a college mathematics classroom (Amstelveen, 2019). In the search string, we did not include the word “engagement” because researchers might have focused on some relevant aspects of engagement (e.g., satisfaction), but did not explicitly use this word (Bond, 2020). The current search string could therefore retrieve all potentially relevant articles with or without using the word “engagement” in their title, abstract, and keywords. The search was run on January 7, 2021.

### Inclusion and Exclusion Criteria

Empirical studies published between January 2011 and December 2020 (10 years) were reviewed. This period covered the majority of the existing flipped classroom research because few studies were published before 2012 (Lo and Hwang, 2018). To be included in this review, the studies had to focus on the use of the flipped classroom approach in mathematics education contexts, such as in teaching algebra, calculus, and statistics. As the aim of this review is to compare student engagement in traditional and flipped classrooms, the included studies should report a comparative between-subjects study. To ensure consistency, the traditional and flipped classrooms involved in the studies should satisfy the aforementioned definitions. In addition, the authors had to compare at least one aspect of student engagement (e.g., attendance and interest) under the two instructional environments. Finally, no constraints were imposed on the language of instruction or location of the studies. However, the manuscripts had to be written in English and published in a peer-reviewed journal because peer review is a useful criterion for including methodologically sound studies.

### Data Extraction and Analysis

The following data were extracted from each article: (a) author(s) of the article and year of publication, (b) country of implementation, (c) course content area, (d) student level, (e) flipped classroom design, and (f) major findings concerning student engagement. Both authors independently extracted the data from the comparative studies. Any discrepancies between the data extracted were reviewed, discussed, and resolved by the authors prior to data entry and analysis.

To answer the research questions, the findings concerning student engagement were analyzed thematically. More specifically, we analyzed quantitative (e.g., surveys) and qualitative (e.g., interviews) data on participants to examine student engagement. As described in the previous section, the general framework for thematic analysis followed the three-dimensional model of student engagement defined by Fredricks et al. (2004), including behavioral engagement (RQ1), emotional engagement (RQ2), and cognitive engagement (RQ3). We examined every comparison item carefully and determined whether the item referred to behavioral, emotional, or cognitive engagement through mutual discussion. To take “Talked about the course contents with peers outside the scheduled hours” (Cronhjort et al., 2018, p. 118) as an example, we categorized this item as belonging to the theme of *interaction* under behavioral engagement. We did so because of the emphasis on students taking action (i.e., behavioral engagement) in exchanging ideas with their classmates.

Ideally, the quantitative results across studies should be summarized using a meta-analytic approach. However, the complex nature of student engagement and the diversity of ways to measure it hindered our attempt to conduct a meta-analysis of the included studies, as Bond (2020) also noted previously. Therefore, following Quin (2017), we calculated effect sizes to determine the strength of the experimental effect when sufficient data (e.g., mean and standard deviation) were reported. The following formulas, as provided by Cohen (1988), were used.

$$\mathrm{Cohen}’s\;d\;=\;\frac{M_{\mathrm{FC}}\;-\;M_{\mathrm{TC}}}{SD_{\mathrm{pooled}}}$$

where

$$SD_{\mathrm{pooled}}\;=\;\sqrt{\frac{SD_{\mathrm{FC}}{}^{2}\;+\;SD_{\mathrm{TC}}{}^{2}}{2}}$$

A positive value of *d* implies that the mean for the flipped class (FC; i.e., the experimental group) is greater than that for the traditional class (TC; i.e., the control group), whereas a negative value implies the opposite. After that, we sought explanations of the results (e.g., significant, non-significant, and group differences) through content analysis of the findings/results, discussions, and conclusions of the included studies (Creswell and Plano Clark, 2011; Akçayır and Akçayır, 2018).

## Results

### Study Selection

By using the search string, a total of 1,186 peer-reviewed journal articles (published from January 2011 to December 2020) were found as of January 7, 2021 (the time of writing). However, 379 articles were removed due to duplication across databases. To recall, the aim of this review is to examine student engagement in mathematics flipped classrooms compared to their traditional counterparts. Although our search string provided the flexibility to capture a variety of terms used to refer to mathematics flipped classrooms, it also yielded many irrelevant search outcomes (e.g., research about Flipgrid and inverters). Therefore, after scanning their titles and abstracts, many articles were excluded because they were not relevant to the purpose of this review. Consistent with Bond (2020), we were aware that some researchers might evaluate some aspects of student engagement without explicitly mentioning them in their title or abstract. Therefore, the headings and subheadings of the articles were scanned in addition to their titles and abstracts.

**FIGURE 1:**
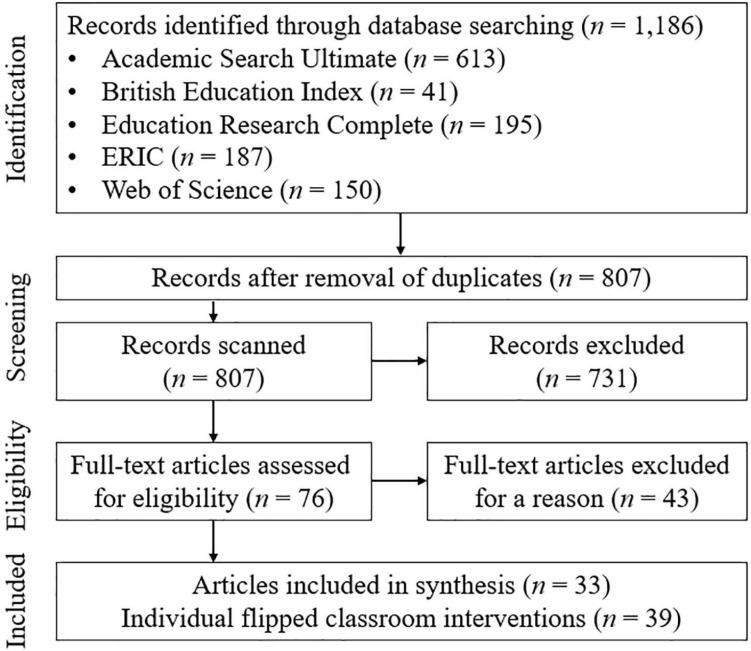
Preferred reporting of items for systematic reviews and meta-analyses flow diagram of article selection.

Ultimately, 76 full-text articles were assessed for eligibility. These articles reported traditional-flipped comparisons in mathematics courses. However, more than half of them were excluded because they did not compare any aspects of student engagement under the two instructional environments. The final selection yielded 33 articles. There was perfect agreement of study selection between the two authors. It is worth noting that Lo and Hew (2020, 2021) reported their intervention in two different articles, and Zack et al. (2015), Hodgson et al. (2017), Rogers et al. (2017) reported more than one flipped course in their articles. Overall, a total of 39 unique individual flipped classroom interventions were involved in this review. Figure 1 outlines the process of article selection.

### Characteristics of the Included Studies

Twenty-four of the 33 included studies (72.7%) were published in the United States. Six studies were from the Asia-Pacific region, including Australia (Khan and Watson, 2018), China (Li et al., 2017; Wang et al., 2020), Hong Kong (Lo and Hew, 2020, 2021), and Taiwan (Bhagat et al., 2016). Three studies were from European countries, including Spain (López Belmonte et al., 2019), Sweden (Cronhjort et al., 2018), and the United Kingdom (Price and Walker, 2021). Except for the study of MBA students by Li et al. (2017), all studies were conducted at the undergraduate (*n* = 25) or secondary school (*n* = 7) level. In the 39 flipped classroom interventions, various content areas of mathematics were involved. At the undergraduate level, the three major content areas were statistics (*n* = 11), calculus (*n* = 9), and algebra (*n* = 4). At the secondary school level, most of the flipped courses (*n* = 5) introduced general mathematics. The background information and the major findings of the included studies are summarized in the Table A1.

To examine student engagement in mathematics courses, almost all included studies (*n* = 29) used student self-report surveys. Several researchers (e.g. Gundlach et al., 2015; Dove and Dove, 2017) adopted some established survey instruments, such as the Survey of Attitudes Toward Statistics-36 (SATS-36; Vanhoof et al., 2011) and the Math Anxiety Rating Scale—Revised (MARS-R; Hopko, 2003). Other researchers developed their own surveys (e.g., Lo and Hew, 2021) or analyzed the results of their course evaluations (e.g., Peterson, 2016). In addition, some researchers conducted classroom observations (e.g., Hodgson et al., 2017), instructor interviews (e.g., Khan and Watson, 2018), and student interviews (e.g., Dove and Dove, 2017). Other methods employed in the included studies were attendance records (e.g., Heuett, 2017), individual quizzes (e.g., Nielsen et al., 2018), and optional assignments (e.g., Lo and Hew, 2020).

**FIGURE 2:**
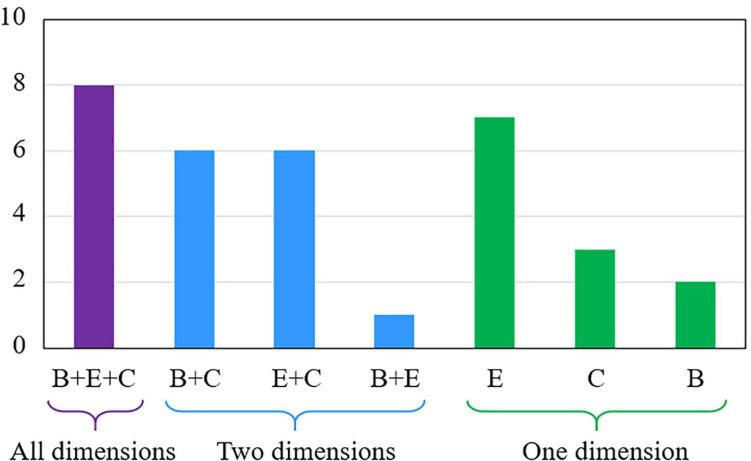
Dimensions of student engagement covered in the included studies. B, Behavioral engagement; E, Emotional engagement; C, Cognitive engagement.

**FIGURE 3:**
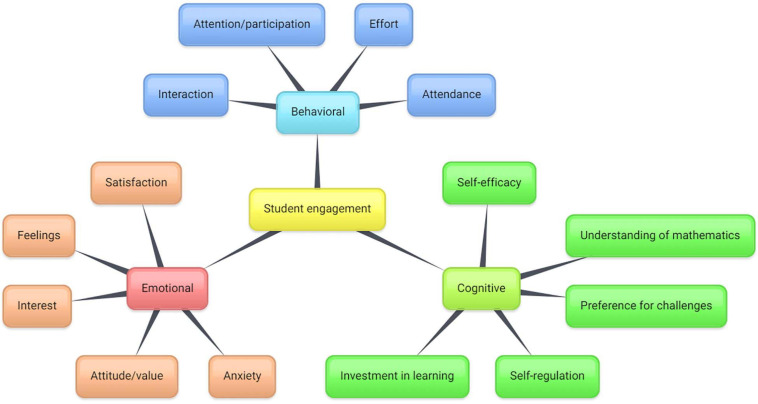
The results of the thematic analysis of the comparison items.

The results of our thematic analysis indicate that only about a quarter of the included studies (*n* = 8) covered all dimensions of student engagement (Figure 2). For example, Price and Walker (2021) compared students’ attendance (i.e., behavioral engagement), level of interest (i.e., emotional engagement), and perceived course difficulty (i.e., cognitive engagement) in their flipped and traditional classes. Overall, we identified 114 comparison items that could be categorized under one of the three dimensions of student engagement. Forty items (35.1%) statistically supported the use of the flipped classroom approach, whereas 11 items (9.6%) were in favor of traditional lecturing. The difference between traditional and flipped classes in 49 items (43.0%) was found to be non-significant. For the remaining 14 items, the data reported in the included studies were not sufficient to determine the significance of the experimental effects statistically. Figure 3 gives an overview of the results of our thematic analysis.

### RQ1: Behavioral Engagement

Thirty-two out of 114 comparison items were related to students’ behavioral engagement. Most of these items could be categorized under three major themes, including interaction (*n* = 13), attention/participation (*n* = 9), and effort (*n* = 7). A few items were concerned with student attendance (*n* = 2) under the two instructional environments. Finally, Lo and Hew (2021) examined students’ overall behavioral engagement (*n* = 1) using the survey by Skinner et al. (2008). Their results suggested that the difference in student ratings between their traditional (*n* = 27, *M* = 4.15, *SD* = 0.45) and flipped (*n* = 28, *M* = 4.02, *SD* = 0.66) classes was not significant, *p* = 0.384. Overall, 12 of the 32 items (37.5%) statistically supported the use of the flipped classroom approach, 13 items (40.6%) were found to be non-significant, and one item (3.1%) was in favor of traditional lecturing. For the remaining six items, the data reported in the included studies were not sufficient to determine the significance of the experimental effects statistically.

First, Table 1 shows that interaction in mathematics courses generally increased significantly after flipping, with effect sizes ranging from *d* = −0.10 to 1.39. For example, Wasserman et al. (2017) found that the use of the flipped classroom approach significantly promoted in-class communication in two consecutive semesters, with a large effect size. Besides the survey items in Table 1, Zack et al. (2015) reported the percentage of students who had a positive perception of instructor–student interaction during class. The differences between the traditional and flipped classes were within 20% in three of their courses, namely business calculus (TC = 91.3% vs. FC = 73.3%), calculus 1 (TC = 72.7% vs. FC = 60.0%), and finite math (TC = 60.0% vs. FC = 77.8%). In their precalculus course, however, only 16.7% of the students in the flipped class rated it positively, compared to 76.5% in the traditional class.

**TABLE 1:** Survey results: Interaction by effect size.

Study	Survey items	*n*_observation_	TC *M* (*SD*)	FC *M* (*SD*)	*p*	*d*
Wasserman et al., 2017	In-class communication (Fall, 2012)	TC = 37, FC = 33	7.53 (2.01)	**10.30 (1.98)**	**0.000**	**1.39**
Wasserman et al., 2017	In-class communication (Spring, 2013)	TC = 30, FC = 33	7.56 (2.04)	**9.46 (2.19)**	**0.001**	**0.90**
López Belmonte et al., 2019	Collaboration	TC = 30, FC = 30	2.30 (0.97)	**2.80 (0.87)**	**0.044**	**0.54**
Wasserman et al., 2017	Outside-of-class peer usefulness (Spring, 2013)	TC = 30, FC = 33	7.72 (2.55)	8.05 (2.21)	0.580	0.14
Cronhjort et al., 2018	Ability to get support from teachers, if needed	TC = 413, FC = 226	3.62	**4.22**	**0.000**	n/a
Cronhjort et al., 2018	Asking peers or teachers when didn’t understand	TC = 413, FC = 226	4.16	**4.54**	**0.000**	n/a
Cronhjort et al., 2018	Learned by working together and discussing with others	TC = 413, FC = 226	4.08	**4.54**	**0.001**	n/a
Cronhjort et al., 2018	Talked about the course contents with peers outside the scheduled hours	TC = 413, FC = 226	4.13	**4.41**	**0.014**	n/a
Wasserman et al., 2017	Outside-of-class peer usefulness (Fall, 2012)	TC = 37, FC = 33	7.08 (2.62)	6.82 (2.68)	0.687	–0.10

*Bold values indicate significant results.*

Second, Table 2 shows that attention/participation in mathematics courses increased significantly in some courses after flipping, with effect sizes ranging from *d* = 0.39 to 0.92. Besides the survey items in Table 2, instructor interviews and classroom observations were conducted in some studies. The tutors in the study by Khan and Watson (2018) reported increased participation in the flipped classroom students’ tutorials compared to those of the traditional classroom students. Through classroom observation, Rogers et al. (2017) analyzed students’ percentage of time-on-task (e.g., taking notes and working). Their results suggested that students’ time-on-task in the flipped class (92.4%) was slightly higher than that in the traditional class (84.1%). Hodgson et al. (2017) also observed student behavior during class. Among their three courses, the observers’ ratings of student attention/participation under different instructional environments were statistically similar in their two algebra courses. Only in their general math course was the rating of their flipped class (*Mdn* = 4.66) significantly higher than that of their traditional class (*Mdn* = 3.50), *p* = 0.011.

**TABLE 2:** Survey results: Attention/participation by effect size.

Study	Survey items	*n*_observation_	TC *M* (*SD*)	FC *M* (*SD*)	*p*	*d*
Bhagat et al., 2016	Attention	TC = 41, FC = 41	2.80 (0.77)	**3.55 (0.86)**	**<0.05**	**0.92**
López Belmonte et al., 2019	Participation	TC = 30, FC = 30	2.00 (0.89)	**2.83 (0.93)**	**0.001**	**0.91**
Amstelveen, 2019	The instructor created opportunities for me to participate in the classroom to support my learning in the course	TC = 20, FC = 29	2.85 (1.35)	3.41 (1.50)	0.188	0.39
Cronhjort et al., 2018	Active participation in the teaching	TC = 413, FC = 226	3.78	4.03	0.104	n/a

*Bold values indicate significant results.*

Third, Table 3 shows that except for Loux et al. (2016), student effort in the traditional and flipped classes was generally similar. For example, Nielsen et al. (2018) designed individual quizzes to measure student effort on the course. Their results suggested that the difference in scores between the traditional and flipped classes was not significant.

**TABLE 3:** Survey results: Effort by effect size.

Study	Survey items	*n*_observation_	TC *M* (*SD*)	FC *M* (*SD*)	*p*	*d*
Loux et al., 2016	Hours spent preparing for class	TC = 52, FC = 45	1.70 (1.42)	**2.50 (2.13)**	**0.016**	**0.44**
Loux et al., 2016	Hours spent completing homework	TC = 52, FC = 45	2.70 (1.80)	**3.30 (2.00)**	**0.046**	**0.32**
Nielsen et al., 2018	Average individual quiz score as a measure of effort	TC = 229, FC = 136	88.04 (7.30)	89.69 (5.65)	>0.05	0.25
Gundlach et al., 2015	Effort: Amount of work the student expends to learn statistics	TC = 193, FC = 25	6.01 (0.88)	6.11 (0.96)	>0.05	0.11
Cronhjort et al., 2018	Worked with the course contents regularly during the course	TC = 413, FC = 226	3.90	4.05	0.351	n/a
Peterson, 2016	Course was demanding	TC = 12, FC = 21	4.50 (0.67)	4.48 (0.68)	0.92	–0.03
Yong et al., 2015	Homework score as a measure of effort	TC = 90, FC = 86	92.43	90.78	>0.05	n/a

*Bold values indicate significant results.*

Finally, two studies compared student attendance under the two instructional environments. Heuett (2017) found that the number of days of absence per student in his flipped class (*M* = 2.60, *SD* = 2.69) was significantly higher than that in his traditional class (*M* = 1.40, *SD* = 1.38), *p* = 0.012. He suggested that this might be “a side-effect resulting from the direct instruction occurring via video outside of class” (p. 895). In contrast, Price and Walker (2021) examined the percentage of students attending seven or more tutorial sessions. Throughout the semester, they observed a downward trend in both the traditional and flipped classes without a significant difference (TC = 74.6% vs. FC = 71.7%), *p* = 0.150.

### RQ2: Emotional Engagement

Forty-one out of 114 comparison items were related to students’ emotional engagement. A majority of these items were categorized under three major themes, including course satisfaction (*n* = 15), feelings (*n* = 10), and interest (*n* = 6). The other items were concerned with students’ attitudes toward and value placed on mathematics (*n* = 5) and anxiety (*n* = 4) under the two instructional environments. Finally, Lo and Hew (2021) examined students’ overall emotional engagement (*n* = 1) using the survey by Skinner et al. (2008). Their results suggested that the difference in student ratings between the traditional (*n* = 27, *M* = 4.16, *SD* = 0.48) and flipped (*n* = 28, *M* = 3.81, *SD* = 0.88) classes was not significant, *p* = 0.132. Overall, 13 of the 41 items (31.7%) statistically supported the use of the flipped classroom approach, 16 items (39.0%) were found to be non-significant, and five items (12.2%) were in favor of traditional lecturing. For the remaining seven items, the data reported in the included studies were not sufficient to determine the significance of the experimental effects statistically.

First, Table 4 shows that except for DeSantis et al. (2015) and Van Sickle (2016), the use of the flipped classroom approach can increase students’ course satisfaction to a certain extent. The effect sizes ranged from *d* = −0.75 to 1.42. Several studies (i.e., Wilson, 2013; Bhagat et al., 2016; Peterson, 2016) even revealed a large effect size in favor of the flipped classroom approach. Besides the survey items in Table 4, Price and Walker (2021) found that 85.2% of their flipped classroom students rated the quality of their lectures as very good or excellent. This percentage was 20% higher than the lecture quality rating for their traditional class (65.1%). In contrast, the percentage of students who expressed satisfaction with the course was the same for traditional (about 73%) and flipped (about 73%) classes in the study by Ziegelmeier and Topaz (2015).

**TABLE 4:** Survey results: Course satisfaction by effect size.

Study	Survey items	*n*_observation_	TC *M* (*SD*)	FC *M* (*SD*)	*p*	*d*
Wilson, 2013	Excellent course	TC = 2 classes of 20 to 25, FC = 2 classes of 20 to 25	3.85 (0.35)	4.40 (0.42)	n/a	1.42
Peterson, 2016	Overall quality of this course	TC = 12, FC = 21	4.00 (0.74)	**4.71 (0.46)**	**<0.01**	**1.15**
Bhagat et al., 2016	Satisfaction	TC = 41, FC = 41	2.96 (0.74)	**3.66 (0.74)**	**<0.05**	**0.95**
Peterson, 2016	Course was a significant contribution	TC = 12, FC = 21	3.83 (0.85)	**4.48 (0.88)**	**0.047**	**0.75**
Peterson, 2016	Course was well organized	TC = 12, FC = 21	4.67 (0.50)	4.81 (0.40)	0.37	0.31
Nielsen et al., 2018	Overall course rating	TC = 208, FC = 130	6.15 (1.25)	6.36 (1.14)	>0.05	0.18
Gundlach et al., 2015	Course rating	TC = 273, FC = 39	4.21 (0.75)	4.31 (0.69)	>0.05	0.14
Li et al., 2017	Course satisfaction score	TC = 45, FC = 75	91.69	**103.42**	**<0.001**	n/a
Touchton, 2015	Course mean	TC = 40, FC = 43	4.27	4.33	>0.05	n/a
Haughton and Kelly, 2015	Rating of course (Spring, 2013)	TC + FC = 231	2.93	3.17	0.08	n/a
Haughton and Kelly, 2015	Rating of course (Fall, 2013)	TC + FC = 250	2.73	2.81	0.98	n/a
Van Sickle, 2016	Overall, I rate the course as excellent	TC = 34, FC = 43	**4.34 (0.74)**	3.95 (1.06)	**0.04**	**–0.43**
DeSantis et al., 2015	Post-lesson feedback survey *[It appeared that the lower the value, the higher the satisfaction]*	TC = 21, FC = 26	**2.36 (0.50)**	2.72 (0.46)	**0.01**	**–0.75**

*Bold values indicate significant results.*

Second, the findings about students’ feelings regarding the flipped classroom approach appear to be mixed across studies. Love et al. (2014) found that significantly more students felt comfortable talking with classmates in their flipped class (about 56%) compared to those in their traditional class (about 21%), *p* = 0.003. According to their pre- and post-survey on student enjoyment, Guerrero et al. (2015) found a positive change in the flipped class but a negative change in the traditional class. However, when comparing the percentage of students reporting a positive feeling about the classroom atmosphere, Zack et al. (2015) found that the effect of the flipped classroom approach was disappointing in some mathematics courses (precalculus: TC = 72.2% vs. FC = 5.6%; business calculus: TC = 73.9% vs. FC = 40.0%; calculus 1: TC = 81.8% vs. FC = 80.0%; finite math: TC = 50.0% vs. FC = 66.7%). Table 5 further shows that the results of the survey items concerning feelings were not in favor of the flipped classroom approach. For example, Zack et al. (2015) examined whether their students would have a dislike of mathematics at the end of their course. The rating for their flipped class was significantly higher (i.e., a higher level of dislike) than that for their traditional class, indicating an inferior effect after flipping.

**TABLE 5:** Survey results: Feelings by effect size.

Study	Survey items	*n*_observation_	TC *M* (*SD*)	FC *M* (*SD*)	*p*	*d*
Zack et al., 2015	The challenge of math appeals to me	TC = 64, FC = 49	2.98	2.57	0.074	n/a
Yong et al., 2015	In this course, I often felt excited about learning new concepts	TC = 90, FC = 86	3.64 (0.81)	3.54 (0.83)	0.624	–0.12
Gundlach et al., 2015	Affect: Students’ feelings concerning statistics	TC = 193, FC = 25	4.92 (0.97)	4.55 (1.28)	>0.05	–0.33
Zack et al., 2015	When I hear the word math, I have a feeling of dislike *[i.e., a smaller value means a better effect]*	TC = 64, FC = 49	**2.62**	3.20	**0.039**	n/a

*Bold values indicate significant results.*

Third, Table 6 shows a mixed finding regarding student interest under different instructional environments. For example, Van Sickle (2016) found that the use of the flipped classroom approach impaired students’ interest in her mathematics course, whereas Haughton and Kelly (2015) and Cronhjort et al. (2018) found the opposite. Besides the survey items in Table 6, Price and Walker (2021) compared the percentage of their traditional and flipped classroom students who found their course interesting. The results were statistically in favor of the flipped classroom approach (TC = 65.7% vs. FC = 84.2%), *p* < 0.05.

**TABLE 6:** Survey results: Interest by effect size.

Study	Survey items	*n*_observation_	TC *M* (*SD*)	FC *M* (*SD*)	*p*	*d*
Cronhjort et al., 2018	Encounter with the assignments that roused interest and engagement	TC = 413, FC = 226	3.47	**3.77**	**0.012**	n/a
Haughton and Kelly, 2015	Raised interest (Spring, 2013)	TC + FC = 231	2.68	**3.26**	**0.05**	n/a
Haughton and Kelly, 2015	Raised interest (Fall, 2013)	TC + FC = 250	2.65	2.63	0.25	n/a
Gundlach et al., 2015	Interest: Students’ level of individual interest in statistics	TC = 193, FC = 25	4.64 (1.19)	4.48 (1.32)	>0.05	–0.13
Van Sickle, 2016	The instructor encouraged my interest in the course	TC = 34, FC = 42	**4.29 (0.71)**	3.79 (1.01)	**0.008**	**–0.57**

*Bold values indicate significant results.*

Finally, a few comparison items for emotional engagement in the included studies were categorized under attitude/value or anxiety. For attitude/value, the results of Li et al. (2017) suggested that their flipped classroom students (*n* = 75, *M* = 60.97, *SD* = 5.33) had a significantly more positive attitude toward cooperative mathematics learning compared to their traditional classroom students (*n* = 45, *M* = 55.18, *SD* = 5.26), with a large effect size of *d* = 1.09, *p* = 0.01. According to their pre- and post-survey on the value placed by students on mathematics, Guerrero et al. (2015) found a positive change in their flipped class but a negative change in their traditional class. However, the results of the studies by Gundlach et al. (2015), Kennedy et al. (2015), and Adams and Dove (2016) suggested that their traditional and flipped classes were statistically similar in terms of students’ attitude/value. For anxiety, Dove and Dove (2017) found that the use of the flipped classroom approach could significantly reduce students’ anxiety about mathematics (TC: *n* = 32, *M*_*post–pre*_ = −6.50, *SD* = 1.70 vs. FC: *n* = 20, *M*_*post–pre*_ = −12.00, *SD* = 1.50) and teaching mathematics (TC: *n* = 20, *M*_*post–pre*_ = −6.00, *SD* = 2.30 vs. FC: *n* = 22, *M*_*post–pre*_ = −9.40, *SD* = 2.00) in their mathematics content courses for pre-service teachers. In Dove and Dove (2015) and Kennedy et al. (2015), however, the difference in students’ anxiety levels between their traditional and flipped classes appeared to be similar.

### RQ3: Cognitive Engagement

Forty-one out of 114 comparison items were related to students’ cognitive engagement. Most of these were categorized under three major themes, including self-efficacy (*n* = 18), understanding (*n* = 12), and preference for challenges (*n* = 6). The other items were concerned with students’ self-regulation (*n* = 2) and investment in learning (*n* = 2). Finally, Lo and Hew (2021) examined students’ overall cognitive engagement (*n* = 1) using the survey by Rotgans and Schmidt (2011). Their results suggested that the difference in student ratings between their traditional (*n* = 27, *M* = 3.75, *SD* = 0.50) and flipped (*n* = 28, *M* = 3.58, *SD* = 0.70) classes was not significant, *p* = 0.304. Overall, 15 of the 41 items (36.6%) statistically supported the use of the flipped classroom approach, 20 items (48.8%) were found to be non-significant, and five items (12.2%) were in favor of traditional lecturing. For the last item, the data reported in the included study were not sufficient to determine the significance of the experimental effects statistically.

First, Table 7 shows a mixed finding about students’ self-efficacy, with effect sizes ranging from *d* = −0.62 to 0.68. The studies by Touchton (2015), Bhagat et al. (2016), López Belmonte et al. (2019), provided evidence that the use of the flipped classroom approach significantly increased students’ self-efficacy (i.e., confidence and self-perceived learning) compared to their counterparts in a traditional class, whereas Gundlach et al. (2015) found the opposite in terms of students’ cognitive competence and the perceived easiness of statistics. The other studies shown in Table 7 suggested that the differences between the two instructional environments were not significant. Besides the survey items in Table 7, Adams and Dove (2016) examined the changes in students’ efficacy, potential for mastery, and perception of difficulty of mathematics; Guerrero et al. (2015) and Loux et al. (2016) examined students’ confidence in mathematics. The results of these studies indicated that the ratings of the traditional and flipped classroom students were statistically similar. In contrast, Heuett (2017) found that his flipped classroom students (about 85%) reported that they had significantly more confidence in their understanding of the course materials compared to his traditional classroom students (about 60%), *p* = 0.030.

**TABLE 7:** Survey results: Self-efficacy by effect size.

Study	Survey items	*n*_observation_	TC *M* (*SD*)	FC *M* (*SD*)	*p*	*d*
Bhagat et al., 2016	Confidence	TC = 41, FC = 41	3.16 (0.69)	**3.64 (0.72)**	**<0.05**	**0.68**
López Belmonte et al., 2019	Overall self-perceived learning	TC = 30, FC = 30	2.14 (0.91)	**2.76 (0.99)**	**0.021**	**0.65**
Wasserman et al., 2017	Resources acquired during course (Spring, 2013)	TC = 30, FC = 33	14.15 (2.98)	14.82 (2.58)	0.341	0.24
Touchton, 2015	Self-assessment of learning in the course	TC = 40, FC = 43	4.30	**4.51**	**<0.01**	n/a
Zack et al., 2015	I believe I am good at solving math problems	TC = 64, FC = 49	3.37	3.09	0.067	n/a
Kennedy et al., 2015	Self-efficacy	TC = 65, FC = 62	40.7 (9.67)	39.8 (9.45)	0.54	–0.09
Yong et al., 2015	I feel well-prepared for the next level of study in this field	TC = 90, FC = 86	3.89 (0.76)	3.82 (0.73)	0.570	–0.09
Wasserman et al., 2017	Resources acquired during course (Fall, 2012)	TC = 37, FC = 33	15.39 (2.46)	14.86 (3.37)	0.458	–0.18
Gundlach et al., 2015	Cognitive competence: Students’ attitudes about their intellectual knowledge and skills when applied to statistics	TC = 193, FC = 25	**5.54 (0.90)**	5.05 (1.20)	**<0.05**	**–0.46**
Gundlach et al., 2015	Perceived easiness: Students’ attitudes about the perceived easiness of statistics as a subject	TC = 193, FC = 25	**4.61 (0.82)**	4.07 (0.93)	**<0.05**	**–0.62**

*Bold values indicate significant results.*

Second, Table 8 shows that except for Zack et al. (2015), the use of the flipped classroom approach can increase students’ understanding of mathematics to a certain extent. The effect sizes ranged from *d* = 0.37 to 0.78. Besides the survey items in Table 8, Murphy et al. (2016) found that their flipped classroom students had a stronger belief that “the underlying mathematical ideas are more important than the formula” (p. 665) than their traditional classroom students. Meanwhile, they rated the proposition that “math is mostly a matter of memorizing formulas and procedures” (p. 665) significantly lower. As the researchers argued, these coupled questions provided evidence that their flipped classroom students finished the course with a better understanding of mathematics. Furthermore, Ziegelmeier and Topaz (2015) found that significantly more students in their flipped class (TC = about 68% vs. FC = about 89%) expressed the opinion that R (a statistical application) helped them to understand their course materials, *p* = 0.028. However, the results of other studies indicated that the students in the two instructional environments were statistically similar in terms of their self-consciousness about mathematics and the utility and importance of mathematics (Adams and Dove, 2016), as well as its perceived relevance to their future career (Love et al., 2014).

**TABLE 8:** Survey results: Understanding of mathematics by effect size.

Study	Survey items	*n*_observation_	TC *M* (*SD*)	FC *M* (*SD*)	*p*	*d*
Peterson, 2016	Clear explanations	TC = 12, FC = 21	4.33 (0.89)	**4.86 (0.36)**	**0.02**	**0.78**
Amstelveen, 2019	Supplementary material helped me learn the course material	TC = 20, FC = 29	2.94 (1.39)	**3.79 (1.37)**	**0.039**	**0.62**
Cronhjort et al., 2018	Related the course contents to real world examples	TC = 413, FC = 226	2.67	**2.94**	**0.048**	n/a
Amstelveen, 2019	Frequent feedback helped me improve my learning in the course	TC = 20, FC = 29	2.95 (1.35)	3.46 (1.4)	0.210	0.37
Cronhjort et al., 2018	The course felt relevant to my on-going studies	TC = 413, FC = 226	4.24	4.38	0.118	n/a
Zack et al., 2015	A strong math background can help me in my professional life	TC = 64, FC = 49	**3.62**	3.25	**0.029**	n/a
Zack et al., 2015	I believe that studying math helps me with problem solving in other areas	TC = 64, FC = 49	**3.57**	3.02	**0.002**	n/a

*Bold values indicate significant results.*

Third, there was evidence that the use of the flipped classroom approach can foster students’ preference for challenges. Compared to their counterparts in a traditional class, the lecturer in the study by Khan and Watson (2018) reported that more students in their flipped class stayed after class to attempt challenging questions. In Lo and Hew (2020), nearly 70% of the students in the flipped class were willing to attempt an optional assignment and challenging questions, whereas fewer than 10% of the students in the traditional class submitted their assignment. Touchton (2015) found that the students in his flipped class (about 58%) were significantly more willing to take additional courses on his content area compared to those in his traditional class (about 26%), *p* < 0.01. Table 9 further shows the results of the survey items about students’ preference for challenges. In contrast to Touchton (2015) and Yong et al. (2015) found that the use of the flipped classroom approach did not affect students’ preference for taking additional courses in their field.

**TABLE 9:** Survey results: Preference for challenges by effect size.

Study	Survey items	*n*_observation_	TC *M* (*SD*)	FC *M* (*SD*)	*p*	*d*
Cronhjort et al., 2018	The course was challenging in a positive way	TC = 413, FC = 226	3.90	**4.32**	**0.000**	n/a
Cronhjort et al., 2018	During the course, worked hard to learn what was difficult	TC = 413, FC = 226	3.75	3.91	0.269	n/a
Yong et al., 2015	I look forward to taking more courses in this field	TC = 90, FC = 86	3.64 (1.00)	3.66 (0.83)	0.895	0.02

*Bold values indicate significant results.*

Finally, a few comparison items for cognitive engagement in the included studies were categorized under self-regulation or investment in learning. For self-regulation, Kennedy et al. (2015) found that the students in their flipped class (*n* = 62, *M* = 243.40, *SD* = 33.07) rated significantly higher than the students in their traditional class (*n* = 65, *M* = 228.90, *SD* = 33.06) in terms of their learning strategies (e.g., rehearsal and elaboration), with a small effect size *d* = 0.44, *p* = 0.02. However, Wang et al. (2020) found that the difference between the traditional (*n* = 44, *M* = 33.25, *SD* = 5.33) and flipped (*n* = 44, *M* = 34.32, *SD* = 5.08) classes was not significant in terms of students’ self-regulated learning, *p* = 0.71. For investment in learning, Cronhjort et al. (2018) used two survey items to examine the difference between their tradition and flipped classes: “Motivated to really learn to understand the course contents” and “When studying, tried to understand how things are connected” (p. 118). Students’ ratings of the former item were significantly in favor of the flipped classroom approach (*M*_*TC*_ = 3.70 vs. *M*_*FC*_ = 4.16, *p* < 0.001), whereas the difference in the latter item was not significant (*M*_*TC*_ = 4.22 vs. *M*_*FC*_ = 4.28, *p* = 0.744).

## Discussion

This section discusses the results of our research synthesis and their implications for future practice and research.

### Behavioral Engagement: Increased Interaction and Attention/Participation but Similar for Effort

We identified three major aspects of measuring behavioral engagement in the included studies, namely interaction, attention/participation, and effort. Most studies provided evidence that the use of the flipped classroom approach increased students’ interaction and attention/participation compared to traditional lecturing. The classroom observation in Rogers et al. (2017) could advance our understanding of such an increase, as the researchers quantified the proportion of class time spent on different instructional activities under the two instructional environments. They found that the students in their traditional class spent most of their class time taking notes (42.6%) and listening (32.0%). In contrast, the students in their flipped class took notes while watching pre-class instructional videos in their individual learning space (Flipped Learning Network, 2014). More class time could thus be spent working on mathematical tasks (35.1%). Most importantly, the percentage of time spent on peer-to-peer collaboration increased from 1.3 to 15.5% after flipping. As echoed by other studies (e.g., Wasserman et al., 2017; Cronhjort et al., 2018; Khan and Watson, 2018), these problem-solving activities not only facilitated instructor–student and student–student interaction but also better supported their attention and participation in the classroom.

Notwithstanding the increase in interaction and attention/participation, students’ levels of effort appeared to be similar in traditional and flipped classrooms across studies. However, we are cautiously positive regarding this non-significant result. As Peterson (2016) commented, “the students in the flipped section did not find the course to be any more demanding than the lecture (traditional) section” (p. 13). Due to the amount of work required, some flipped classroom interventions overwhelmed and frustrated students (Lo et al., 2017; van Alten et al., 2019; Bond, 2020). In the words of one student, “I felt as if I didn’t have enough time to finish what I needed, so I felt rushed. For this reason, I didn’t really enjoy the flip” (Moran, 2018, p. 11). Therefore, instructors should maintain a similar course workload when transforming their traditional lecture-based mathematics course into a flipped one. As Wasserman et al. (2017) specified, the total time required for students to complete out-of-class work for flipped classrooms (e.g., class preparation) should be approximately the same as for traditional classrooms (e.g., homework).

### Emotional Engagement: Increased Satisfaction but Mixed Results for Feelings and Interest

We identified three major aspects of measuring emotional engagement in the included studies, namely course satisfaction, feelings, and interest. Most studies supported the idea that the use of the flipped classroom approach increases students’ course satisfaction compared to traditional lecturing. This result is not consistent with the review by van Alten et al. (2019). The researchers conducted a meta-analysis of 22 studies across contexts and subject disciplines and found that the overall effect size of the flipped classroom approach was negligible and non-significant (Hedges’ *g* = 0.05, 95% CI = [−0.23, 0.32], *p* = 0.73). In particular, more than half of their included studies reported a negative or neutral effect, whereas this review found the opposite in mathematics education. For example, Wilson (2013), Bhagat et al. (2016), and Peterson (2016), found that students’ rating of course satisfaction in their flipped class was significantly higher than that in their traditional class, with a large effect size. Bhagat et al. (2016) explained that their flipped classroom students could access their pre-class learning materials at a time convenient to them, which was not possible for those in their traditional classroom. Inside the classroom, their students, especially those who were underperforming, could receive more support from their instructor and peers because the class time was no longer occupied by direct instruction. As Peterson (2016) concluded, the instructional sequence of the flipped classroom approach caused the difference indicated in course satisfaction.

Despite the increased course satisfaction, the results regarding students’ feelings and interests were mixed. The studies by Zack et al. (2015) and Van Sickle (2016) even provided evidence that the use of the flipped classroom approach could have a negative impact on these two aspects. Van Sickle (2016) explained that the students in her flipped class often had a hard time during class meetings because the in-class work problems were difficult. This negative experience thus impaired their emotional engagement. In their reflection, Zack et al. (2015) suggested designing a wide variety of in-class activities to ensure student engagement during class. Similarly, Cronhjort et al. (2018) emphasized the value of using a broad range of learning activities in their flipped classroom. At the start of lessons, they would first ensure that students had adequate preparation for handling more advanced learning tasks by offering instructor-led reviews. After that, they used multiple-choice questions (easy), hands-on exercises (medium), and applied problems (hard) to guide student learning. As a result, their flipped classroom students had a higher level of interest in the learning tasks compared to their traditional classroom students. In future practice, instructors should design a sequence of in-class learning activities for students to develop the desired mathematical knowledge and skills progressively.

### Cognitive Engagement: Mixed Results for Self-Efficacy but Increased Understanding of Mathematics and Preference for Challenges

We identified three major aspects of measuring cognitive engagement in the included studies, namely self-efficacy, understanding of mathematics, and preference for challenges. The results regarding students’ self-efficacy were mixed across studies. We found that the use of the flipped classroom approach in Gundlach et al. (2015) produced the least favorable effect. One possible reason for such a decrease might be the reduction of class time after flipping. The students in the traditional class would meet their instructor or teaching assistant three times a week (a total of 150 mins) but only once a week (a total of 75 mins) in the flipped class. Gundlach et al. (2015) thus lamented that the instructor’s formative feedback opportunities were sacrificed. In contrast, Touchton (2015) was able to provide students with immediate feedback during class. He argued that the feedback could prevent students from acquiring bad habits in solving statistical problems. As a result, the students in his flipped class had greater confidence in applying their knowledge compared to those in his traditional class. From the perspective of self-determination theory, timely feedback is essential to increase students’ self-efficacy and thus their cognitive engagement (Fredricks et al., 2004; Niemiec and Ryan, 2009).

Despite the mixed results regarding self-efficacy, most studies provided evidence that the use of the flipped classroom approach increased students’ understanding of mathematics and fostered their preference for challenges. We found that the interventions with a positive effect (e.g., Touchton, 2015; Ziegelmeier and Topaz, 2015; Murphy et al., 2016; Cronhjort et al., 2018; Lo and Hew, 2020) generally emphasized the real-world applications of course materials. For example, Lo and Hew (2020) used their class time better after flipping by engaging students in solving real-world problems. In an optional learning task in their course, most students in their flipped class were willing to attempt challenging questions, whereas those in their traditional class were not. Touchton (2015) designed his learning tasks using authentic examples, such as the statistical models presented in journal articles. With more class time devoted to applying what they had learned in their course, his flipped classroom students were better able to master and understand the value of the course materials. Therefore, they had a greater willingness to pursue additional training compared to his traditional classroom students.

To summarize, the results of our research synthesis have the following implications for future flipped classroom practice to support student engagement. These implications are related to the design features (i.e., retaining student workload and facilitating collaborative problem-solving) and elements (i.e., the use of in-class review, real-world problems, and instructor feedback) of mathematics flipped classrooms.

(1) Student workload: Retaining the same level of course workload when transforming a traditional lecture-based course into a flipped one.
(2) In-class review: Offering instructor-led reviews at the start of lessons to ensure students’ readiness to handle advanced problems.
(3) Collaborative problem-solving: Engaging students in solving a progression of problems with peer support.
(4) Real-world problems: Enabling students to appreciate the usefulness of course materials by using real-world mathematical problems.
(5) Instructor feedback: Using class time to provide feedback on student performance and clarify their misunderstandings.

### Limitations and Implications for Future Research

Although this review can contribute to our understanding of student engagement in mathematics flipped classrooms, several limitations must be acknowledged. These limitations have important implications for future research regarding the research contexts, focuses, and methods. First, the majority of the comparative studies of student engagement were conducted in the United States and in higher education contexts. Therefore, the results of this review may not be generalizable to other contexts. Further research is required to examine the effect of the flipped classroom approach on non-United States (e.g., Asian) and/or K–12 students’ engagement in mathematics courses.

Second, this review discovered quite a few aspects of student engagement that have not been thoroughly explored. For behavioral engagement, only two included studies (i.e., Heuett, 2017; Price and Walker, 2021) compared students’ attendance in their traditional and flipped classes. For emotional engagement, only three included studies (i.e., Dove and Dove, 2015, 2017; Kennedy et al., 2015) focused on mathematics anxiety. In particular, the usefulness of the findings of Dove and Dove (2015, 2017) may be confined to mathematics courses for pre-service teachers. For cognitive engagement, only two included studies (i.e., Kennedy et al., 2015; Wang et al., 2020) examined students’ self-regulation in their flipped classes compared to their traditional classes. Due to the limited number of studies, conclusions cannot be drawn about students’ attendance, mathematics anxiety, self-regulation, or other minor themes (e.g., attitude/value and investment in learning). Future research could focus on these aspects and evaluate the effect of the flipped classroom approach.

Third, most of the included studies used self-report surveys to evaluate student engagement with their mathematics courses. However, the survey items were diverse across studies. As noted previously by Bond (2020), such a diversity of measurements hinders further quantitative analysis (e.g., meta-analysis) of student engagement. Therefore, we suggest using some established survey instruments (e.g., Gundlach et al., 2015; Dove and Dove, 2017) or developing the instruments based on the literature on student engagement (e.g., Lo and Hew, 2021) in future research, which will enable a better comparison and synthesis across studies. Besides, the results of this review were largely based on the self-report survey data of the included studies. However, the results of these self-report data should be viewed with caution because the research participants may have provided socially desirable responses. Therefore, more objective measurements of student engagement should be used in future research, such as classroom observation (to monitor students’ behavior) and optional assignments (to evaluate students’ preference for challenges).

## Conclusion

This review focused on student engagement in mathematics courses under different instructional approaches. Thirty-three traditional-flipped comparative studies were analyzed. The results suggested that the use of the flipped classroom approach could increase certain aspects of behavioral engagement (i.e., interaction and attention/participation), emotional engagement (i.e., satisfaction), and cognitive engagement (i.e., understanding of mathematics and preference for challenges). However, the results with respect to a few aspects of emotional engagement (i.e., feelings and interest) and cognitive engagement (i.e., self-efficacy) appeared to be mixed across studies. Based on the results of our research synthesis, several recommendations for future practice and research were made. This review thus contributed to our understanding of (1) the effect of using the flipped classroom approach on student engagement in mathematics education, (2) how to better support student engagement in future flipped classroom practice, and (3) possible directions for further research on flipped classrooms.

## Data Availability Statement

The raw data supporting the conclusions of this article will be made available by the authors, without undue reservation.

## Author Contributions

CL performed the analysis and wrote the first draft. KH helped to revise the manuscript. Both authors contributed to the design of the study, data collection, data coding, and read and approved the final manuscript.

## Conflict of Interest

The authors declare that the research was conducted in the absence of any commercial or financial relationships that could be construed as a potential conflict of interest.

## Appendix

**TABLE A1:** This appendix provides a summary of the background information and the major findings of the included studies.

Study	Location	Subject area (Grade level)	Major findings
Adams and Dove, 2016	United States	Calculus (UG)	The flipped classroom approach was not found to have any significant impact on students compared to traditional lecturing. However, the flipped classroom students showed appreciation for this new instructional approach and wished to take more mathematics courses using it.
Amstelveen, 2019	United States	Algebra (UG)	There were no significant differences on in-class exams between the traditional and flipped classes. However, the students in the flipped class perceived that the video lectures helped them to learn more mathematics compared to those in the traditional class.
Bhagat et al., 2016	Taiwan	Trigonometry (SS)	The flipped classroom students had significantly higher learning achievement and motivation than the traditional classroom students. The performance of low achievers in the flipped class was better than those in the traditional class.
Cronhjort et al., 2018	Sweden	Calculus (UG)	Compared to the traditional class, the normalized learning gain was 13% higher in the flipped class. Besides, the flipped classroom students rated significantly higher on their engagement survey.
DeSantis et al., 2015	United States	Geometry (SS)	There were no significant differences in the learning outcomes between the traditional and flipped classes. However, the students in the traditional class reported significantly higher satisfaction with their own learning than those in the flipped class.
Dove and Dove, 2015	United States	Math content course for pre-service teachers (UG)	The mathematics anxiety scores decreased significantly in both the traditional and flipped classes. However, the flipped classroom students achieved significantly higher in the overall course grades.
Dove and Dove, 2017	United States	Math content course for pre-service teachers (UG)	Students’ anxieties related to mathematics were improved in both the traditional and flipped classes. In the flipped class, teacher-created videos better aligned with course content and activities. Therefore, the students felt prepared and more confident before entering the classroom.
Guerrero et al., 2015	United States	Finite math (UG)	The use of the flipped classroom approach had positive effects on student attitudes toward mathematics. However, the new instructional approach had no significant impact on student learning over traditional lecturing.
Gundlach et al., 2015	United States	Statistics literacy (UG)	The traditional classroom students scored higher on average on all three exams compared to the flipped classroom students. However, their differences in homework, projects, and university evaluations of the course and instructor were not significant.
Haughton and Kelly, 2015	United States	Statistics (UG)	The flipped classroom students performed better than the traditional classroom students on the common final exam. However, there were no significant differences in the final grades and student satisfaction between the traditional and flipped classes.
Heuett, 2017	United States	Statistics (UG)	The flipped classroom students performed better than the traditional classroom students on exams. Moreover, they were more confident about their abilities and their understanding of the course materials.
Hodgson et al., 2017	United States	Algebra (SS); Algebra (SS); General math (SS)	An increase in engagement was found in only one of the three flipped classes. Student engagement was affected by student characteristics and instructors’ skills and expectations.
Kennedy et al., 2015	United States	Calculus (UG)	The students in the traditional class significantly outperformed those in the flipped class on conceptual portions of some exams. Moreover, the overall motivation score for the flipped classroom students significantly dropped from the pre-test to the post-test. Nevertheless, there was an increase in both the rehearsal score and peer learning score for the flipped classroom students.
Khan and Watson, 2018	Australia	Statistics (UG)	The use of the flipped classroom approach improved student performance, understanding of concepts, and student engagement. The findings of student feedback indicated a higher preference for the flipped classroom approach, especially for ages 20 and below.
Li et al., 2017	China	Statistics (MBA)	There were no significant differences in learning achievement, course satisfaction, and cooperative learning attitudes between the traditional and flipped classes. Web-based learning self-efficacy influenced their learning achievement and course satisfaction.
Lo and Hew, 2020, 2021	Hong Kong	General math (SS)	Compared to traditional lecturing, the use of the flipped classroom approach with gamification improved students’ learning achievement and their submission rate of an optional assignment (i.e., their preference for challenges). However, the survey results indicated that students’ behavioral, emotional, and cognitive engagement were similar under different instructional environments.
López Belmonte et al., 2019	Spain	General math (SS)	The use of the flipped classroom approach improved several attitudinal (including motivation, autonomy, collaboration, and participation) and mathematical (including scientific data, graphics, results, and decision) dimensions compared to traditional lecturing.
Loux et al., 2016	United States	Statistics (UG)	The flipped classroom students reported very high satisfaction with the instructional approach. However, students’ end-of-semester opinions and levels of confidence were similar in the traditional and flipped classes.
Love et al., 2014	United States	Algebra (UG)	The students in the flipped class experienced a more significant increase between the sequential exams compared to those in the traditional class. However, they performed similarly in the final exam. The flipped classroom students were very positive about their experience in the course and appreciated the student collaboration and instructional video components.
Murphy et al., 2016	United States	Algebra (UG)	The flipped classroom students performed better in the overall comprehension of content with a 21% increase in the median final exam score. They felt more confident in their ability to learn mathematics independently, showed better retention of materials over time, and enjoyed the experience in the flipped classroom.
Nielsen et al., 2018	United States	Statistics (UG)	The use of the flipped classroom approach significantly improved student performance and course satisfaction. With the help of teaching assistants and the use of additional classrooms, the instructional approach could be used in large lecture classes.
Peterson, 2016	United States	Statistics (UG)	The students in the flipped class outperformed those in the traditional class by more than a letter grade on the final exam. The flipped classroom students were more satisfied with the course overall. These results were likely due to the strong cohesion between the in-class and out-of-class content.
Price and Walker, 2021	United Kingdom	Statistics (UG)	There were no significant differences in exam performance, class attendance, and online engagement between the traditional and flipped classes. Student perceptions of the flipped classroom approach differed according to gender, nationality, and reported prior mathematics training.
Rogers et al., 2017	United States	Algebra (SS); Pre-AP (SS); General math (SS)	Student engagement in the flipped classes was higher than that in the traditional classes. The flipped classroom students spent more class time working on mathematics topics and collaborating with peers, whereas the traditional classroom students spent more time taking notes.
Touchton, 2015	United States	Statistics (UG)	The flipped classroom approach gave students a statistically significant advantage in difficult and applied areas emphasized in class. The students in the flipped class expressed that they learned more and enjoyed the course more than those in the traditional class.
Van Sickle, 2016	United States	Algebra (UG)	The flipped classroom students scored higher than the traditional classroom students in the final exam. However, their perception of several measures decreased significantly, including how interested they were in the course and whether the instructor effectively facilitated learning.
Wasserman et al., 2017	United States	Calculus (UG)	Student performance on procedural problems was similar in the traditional and flipped classes. The flipped classroom students reported increased communication during class, but the traditional classroom students perceived more effective use of class time.
Wilson, 2013	United States	Statistics (UG)	The use of the flipped classroom approach freed up more class time for interactive activities. It had a positive impact on students’ attitudes toward the class and instructor as well as on their performance.
Yong et al., 2015	United States	Calculus (UG)	The differences in learning, metacognitive, and affective gains between the traditional and flipped classes were not significant. These results were likely due to contextual factors (e.g., a strong group-work culture) in the research site.
Zack et al., 2015	United States	Finite math (UG); calculus (UG); calculus (UG); calculus (UG)	No statistical difference was found in the test scores of the traditional and flipped classroom students. However, many flipped classroom students had negative opinions of the new instructional approach and their attitudes toward mathematics tended to decline in general.
Ziegelmeier and Topaz, 2015	United States	Calculus (UG)	The traditional and flipped classroom students scored similarly on the graded components of the course. Moreover, the majority of students in both classes were comfortable with the course format.

*SS, Secondary school; UG, Undergraduate.*
